# Supplementary material for: Evaluation of the design and implementation of municipal ice cleat distribution programs for the prevention of ice-related fall injuries among older adults in Sweden
Source: PLoS One. 2021 Jun 25;16(6):e0253054. doi: 10.1371/journal.pone.0253054 (PMC8232537; doi:10.1371/journal.pone.0253054)
Supplement: S1 Appendix — (DOCX) [file pone.0253054.s001.docx]

Semi-structured interview guide

Inform the participants about the purposes of the study, informed consent, etc.

**Battery 1: Questions about risk of falling in winter**

- Do you use any anti-slip protection?
- What do you think about the upcoming winter? (Is that something you worry about?)
- What is it like to use ice cleats?
- How do you think about the risk of falling in the winter?
- What affects if you use anti-slip protection?
- What can prevent you from using anti-slip protection?
- What could make you use anti-slip protection?

**Battery 2: About falls**

- What consequences do you think a fall can have?
- Can you tell us about times when you fell?
- Can you then tell us what you think about the risk of falling?
- Have you been injured by a fall?
- Are you afraid of falling?
- Have you seen others fall?
- Has anyone near you been injured in a fall?

**Battery 3: Economic aspects**

- Have you purchased any anti-slip protection?
- Does the cost matter?
- Do you think that anti-slip protection is expensive?
- How much can you imagine paying?

**Battery 4: Ice cleat distribution programs**

- Do you think it would be possible to design an intervention to get people to use anti-slip protection?
- Some municipalities have distributed ice cleats and we are trying to investigate whether this type of measure has an effect. How would you react to being offered free ice cleats?
- What type of subsidy would you prefer? That you are provided with a set of ice cleats, which you get financial support to buy them on you own, or do you have any other suggestions?
- Ask if they have suggestions for designing these programs.

**Battery 5: Physical activity**

- Do you usually exercise?
- Do you usually go out for a walk?
- Does the weather affect whether you go out or not?

**Battery 6: Risk perception**

- Do you think about the risk of falling and hurting yourself?
- How do you think about other risks in your life?
- What are you worried about?
- Can you tell us about risks in your everyday life?
- What about other societal risks?
