# Supplementary material for: Evaluation of the design and implementation of municipal ice cleat distribution programs for the prevention of ice-related fall injuries among older adults in Sweden
Source: PLoS One. 2021 Jun 25;16(6):e0253054. doi: 10.1371/journal.pone.0253054 (PMC8232537; doi:10.1371/journal.pone.0253054)
Supplement: S1 Table — (DOCX) [file pone.0253054.s002.docx]

**Supplementary Table S1. Key components of the proposed logic model for ice cleat distribution programs, with theoretical arguments, counterarguments and sources of evidence.**

| **Model component** | **Key theoretical argument** | **Potential issues / counterargument** | **Source of evidence (full references provided at the bottom)** | **Comment** |
| --- | --- | --- | --- | --- |
| Political support and formal decision to implement municipal program. | In a Swedish municipal context, there must be local political support to implement an ice cleat program. | The program is not implemented due to lack of political support, lack of resources, no scientific evidence, or no evidence of cost-effectiveness. | Local Government Act [1], public decision documents, survey data. | According to the Local Government Act, all municipalities are self-governing. In short, they make their own decisions on whether ice cleat programs are introduced. Survey data provides some insight into why some municipalities have chosen to introduce programs. Public decision documents display the municipal decision-making process and explicitly show the reasons why some municipalities have chosen to introduce programs and others have not. |
| Resources. | The resources that go into the program can affect the reach and impact of the program. | Improper, or insufficient, resources in terms of money, the number of purchased ice cleats, the total of staff members, and their know-how (i.e. their ability to establish distribution and communication plans, and facilitate the program intervention accordingly) can affect program implementation and program reach negatively.  External factors may also affect the program negatively, such as ice cleats get stuck in customs. | Rossi et al. [2], Saunders et al. [3], (U. Stefansson, personal communication, August 8, 2019), survey data. | Survey data made it clear that resources were crucial for the implementation of the programs. This is also emphasized in the program evaluation literature. |
| Distribution plan. | Together with the communication plan, a well-executed distribution plan will ease the hand out of the ice cleats. | Limiting distribution points and/or limiting the time frame of the ice cleats that can be picked up can adversely affect program reach. | Rossi et al. [2], Bonander & Holmberg [4], survey data. | Survey data show bad examples of limiting time and distribution point can have an adverse effect on reach. An earlier study of a Swedish ice cleat program shows that distributing pick-up locations throughout the city can make it easier for the target population to pick up a pair. |
| Communication plan. | Together with the distribution plan, the realization of the communication plan will create awareness about the program to the targeted population. | Poorly executed communication plan can affect the targeted populations’ awareness about the program. | Aldoory & Bonzo [5], Saunders et.al. [3], Rossi et al. [2], Bonander & Holmberg [4], Bandura [6], Champion et al. [7], survey data, interview data. | It is imperative to create awareness about the program. If the targeted population does not know of an existing program, it is doomed to fail. A quasi-experimental study indicates that sending coupons home to the target population can be a possible way to create awareness about the program. According to Aldoory & Bonzo, multichannel campaigns can reach a wider range of the target population. |
| Ice cleats distributed to a meaningful share of the target population. | Properly executed communication and distributions plans will affect the targeted population to retrieve ice cleats. | A milder or warmer winter can affect the target population not to pick up a couple of ice cleats.  Time limit for picking up a pair of ice cleats can affect reach negatively.  Individuals may already own a pair of ice cleats and thus not pick up a pair of ice cleats. These group of individuals contribute to a ceiling effect. | Aldoory & Bonzo [5], Bonander & Holmberg [4], Gustavsson et al. [8], Gielen & Sleet [9], survey data, interview data. | A quasi-experimental study suggests that spreading out distribution points throughout the city generates greater program reach.  Our survey data indicates that the amount of purchased ice cleats have an effect on the amount of ice cleats distributed. One study points out that about 40% of the population above 65 reports seldom or never use of ice cleats, suggesting that a significant part of the targeted population can be reached and thus become “new” ice cleat users. |
| Increase in ice cleat use during icy road conditions as an effect of the program | By providing the target population with a pair of ice cleats, there will be an increased number of ice cleat users. | Given the diversity of ice cleats designs, some ice cleats may be unsuitable for some individuals (according to their preferences) and thus not be used.  An initial poor user experience may adversely affect future ice cleat use.  Ice cleats can easily brake or deteriorate over time.  Behavior post-implementation appears to be difficult to uphold and potential behavior changes in compliance with the program can be expected to be short-lived. | Bonander & Holmberg [4], Gard & Lundborg [10, 11], Berggård & Johansson [12], Champion et al. [7], Kwasnicka et al. [13], McAuley [14], Aldory & Bonzo [5], Gielen & Sleet [9], Bandura [6], interview data. | Banduras theories of self-efficacy, together with the target population’s perceived benefits of the program, suggest that ice cleat users may increase. Despite this, there are studies that suggest that behavior change may be short-lived, and the use of ice cleats may be limited to the introductory year. Previous research and interview data suggest that ice cleats need to be user friendly in order to apply them to the feet.  See Focus-group interviews in the results section. |
| Reduction in ice-related fall injuries | Ice cleats increase friction and should, all else being equal, reduce fall risk when walking on icy surfaces. | May increase risk exposure via increased walking or by risk compensation (e.g., individuals may not avoid icy spots as much as they would have without ice cleats). | McKiernan [15], Gard & Lundborg [10, 11], Gard & Berggard [16], Bruce et al. [17], Merrild & Bak [18], Bonander & Holmberg [4], interview data. | One randomized experiment indicates a reduction in injury risks when ice cleats were used. A quasi-experimental study of a Swedish municipal program show a reduction of ice-related fall injuries the introductory year.  See Focus-group interviews in the results section. |
| Increased walking during icy conditions. | Access to ice cleats may decrease fear of falling during winter conditions. | Ice cleats may primarily affect behaviors among older adults who already have strong intention to be active.  The use of ice cleats may create a sense of false security. This makes individuals less careful when walking over icy parts. | McKiernan [15], Berggård & Johansson [12], interview data. | Participants from a randomized experiment, and a Swedish study show increase of walking when using ice cleats.  See Focus-group interviews in the results section. |
| Improved health and well-being. | The access to ice cleats may spur the targeted population to be more active outdoors when winter conditions are prevalent. This can contribute to an improved public health. | Individuals who do not see walking as health-beneficial may use other ways of transport.  Bad experiences of ice cleats use can make individuals stay indoors. | Lee & Buchner [19], Kerr et al. [20], Faskunger [21], Gard & Lundborg [10, 11]. | Two independent studies suggest that active transports, such as walking, can contribute to health benefits. Physical activity is also effective to prevent pedestrian falls and fall-related injuries among older adults. |

# References

1. Kommunallag (2017:725).

2. Rossi HP, Lipsey WM, Henry TG. Evaluation A Systematic Approach. London: Sage; 2019.

3. Saunders RP, Evans MH, Joshi P. Developing a process-evaluation plan for assessing health promotion program implementation: a how-to guide. Health Promot Pract. 2005;6(2):134-47. Epub 2005/04/28. doi: 10.1177/1524839904273387. PubMed PMID: 15855283.

4. Bonander C, Holmberg R. Estimating the effects of a studded footwear subsidy program on pedestrian falls among older adults in Gothenburg, Sweden. Accid Anal Prev. 2019;132:105282. Epub 2019/09/21. doi: 10.1016/j.aap.2019.105282. PubMed PMID: 31539867.

5. Aldoory L, Bonzo S. Using communication theory in injury prevention campaigns. Injury Prevention. 2005;11(5):260-3. doi: 10.1136/ip.2004.007104.

6. Bandura A. Self-efficacy: The exercise of control. New York, NY, US: W H Freeman/Times Books/ Henry Holt & Co; 1997.

7. Champion LV, Skinner CS. The health belief model. In: Glanz K, Rimer KB, K V, editors. Health Behavior and Health Education: Theory, Research, and Practice. 4th ed. San Fransisco CA: Jossey-Bass; 2008. p. 45-65.

8. Gustavsson J, Nilson F, Bonander C. Individual and contextual factors associated with the use of anti-slip devices according to a Swedish national survey. Journal of Transport & Health. 2020;17:100865. doi: https://doi.org/10.1016/j.jth.2020.100865.

9. Gielen AC, Sleet D. Application of Behavior-Change Theories and Methods to Injury Prevention. Epidemiologic Reviews. 2003;25(1):65-76. doi: 10.1093/epirev/mxg004.

10. Gard G, Lundborg G. Pedestrians on slippery surfaces during winter--methods to describe the problems and practical tests of anti-skid devices. Accid Anal Prev. 2000;32(3):455-60. Epub 2000/04/25. doi: 10.1016/s0001-4575(99)00070-6. PubMed PMID: 10776864.

11. Gard G, Lundborg G. Test of Swedish anti-skid devices on five different slippery surfaces. Accident Analysis & Prevention. 2001;33(1):1-8. doi: 10.1016/s0001-4575(00)00002-6. PMID: 11189113.

12. Berggård G, Johansson C. Pedestrians in wintertime—Effects of using anti-slip devices. Accident Analysis & Prevention. 2010;42(4):1199-204. doi: https://doi.org/10.1016/j.aap.2010.01.011.

13. Kwasnicka D, Dombrowski SU, White M, Sniehotta F. Theoretical explanations for maintenance of behaviour change: a systematic review of behaviour theories. Health Psychology Review. 2016;10(3):277-96. doi: 10.1080/17437199.2016.1151372.

14. McAuley E. Self-efficacy and the maintenance of exercise participation in older adults. Journal of Behavioral Medicine. 1993;16(1):103-13. doi: 10.1007/BF00844757.

15. McKiernan FE. A simple gait-stabilizing device reduces outdoor falls and nonserious injurious falls in fall-prone older people during the winter. Journal of the American Geriatrics Society. 2005;53(6):943-7. PubMed PMID: edscal.16908376.

16. Gard G, Berggard G. Assessment of anti-slip devices from healthy individuals in different ages walking on slippery surfaces. Applied Ergonomics. 2006;37(2):177-86. Epub 2005/08/24. doi: 10.1016/j.apergo.2005.04.004. PubMed PMID: 16115606.

17. Bruce M, Jones C, Manning DP. Slip-resistance on icy surfaces of shoes, crampons and chains — A new machine. Journal of Occupational Accidents. 1986;7(4):273-83. doi: https://doi.org/10.1016/0376-6349(86)90018-0.

18. Merrild U, Bak S. An excess of pedestrian injuries in icy conditions: A high-risk fracture group—elderly women. Accident Analysis & Prevention. 1983;15(1):41-8. doi: https://doi.org/10.1016/0001-4575(83)90005-2.

19. Lee I-M, Buchner DM. The Importance of Walking to Public Health. Medicine & Science in Sports & Exercise. 2008;40(7):S512-S8. doi: 10.1249/MSS.0b013e31817c65d0. PubMed PMID: 00005768-200807002-00002.

20. Kerr J, Rosenberg D, Frank L. The Role of the Built Environment in Healthy Aging:Community Design, Physical Activity, and Health among Older Adults. Journal of Planning Literature. 2012;27(1):43-60. doi: 10.1177/0885412211415283.

21. Faskunger J. Den byggda miljöns påverkan på fysisk aktivitet: En kunskapssammanställning för regeringsuppdraget “Byggd miljö och fysisk aktivitet”. 2007 (R 2007:3). Statens folkhälsoinstitut. Available from: <https://www.folkhalsomyndigheten.se/contentassets/bd4c8de3a04b4d20ac7e0f0385193663/byggda-miljons-paverkan-fysisk-aktivitet.pdf>
