# Supplementary material for: Evaluation of the design and implementation of municipal ice cleat distribution programs for the prevention of ice-related fall injuries among older adults in Sweden
Source: PLoS One. 2021 Jun 25;16(6):e0253054. doi: 10.1371/journal.pone.0253054 (PMC8232537; doi:10.1371/journal.pone.0253054)
Supplement: Attachment — Submitted filename: Response to Reviewers.docx [file pone.0253054.s003.docx]

Dear Ahmed Mancy Mosa,

Thank you for your valuable feedback. We have incorporated your input into the revised manuscript. We address your comments in the table below.

| **The editor’s comments to the author** | **Authors’ response** |
| --- | --- |
| 1. Please ensure that your manuscript meets PLOS ONE's style requirements, including those for file naming. The PLOS ONE style templates can be found at <https://journals.plos.org/plosone/s/file?id=wjVg/PLOSOne_formatting_sample_main_body.pdf> and <https://journals.plos.org/plosone/s/file?id=ba62/PLOSOne_formatting_sample_title_authors_affiliations.pdf> | **Response to editor:**  Thank you for pointing this out to us. The pdfs help us clarify this.  **Revised text:**  We have now changed the manuscript according to the style templates. We have also changed the file names of the figures; “Fig1.tif” & “Fig2.tif”, and re-uploaded these with its changed names.  According to the manuscript body formatting guidelines, we have moved the supporting information section to the bottom of the manuscript. |
| 2. When reporting the results of qualitative research, we suggest consulting the COREQ guidelines: <http://intqhc.oxfordjournals.org/content/19/6/349>   In this case, please consider including more information on the number of interviewers, their training and characteristics; and please provide and clarify whether an interview guide used.  Furthermore, in the Methods section, please include details regarding the date range of the literature search conducted. | **Response to editor:**  We understand that clarifications are needed here. We upload the pdf named “COREQ_Checklist” to provide clarifications about the interview procedure. We also upload a supplementary appendix showing the used interview guide.  **Revised text:**  We clarify how the participants were approached in line 146-147: “Two independent local associations, with older adults as their target group, were contacted *face-to-face* and asked if they were interested in participating in focus group interviews about the use of ice cleats and municipal ice cleat programs.”  We added the following text in the lines 152-163: “*Prior to the interviews, we prepared a semi-structured interview guide with a battery of questions (see Supplementary Appendix) which was used as guidance during the sessions [14]. The interviews were conducted by one male and one female researcher (RH & JG). JG has a long background in health care, is a registered nurse, has a Ph.D. in risk and environmental studies, and is specialized in fall-preventive measures in the elderly. JG also has extensive experience in the field of qualitative interviews for research purposes. JG acted as the main interviewer and moderator of the sessions. RH is a Ph.D.-student and had limited prior experience in conducting interviews. Therefore, RH acted as a moderator assistant by managing the logistics, monitoring the recording equipment, and taking careful notes.* *One session was held at the gym and the other at the bridge club. No other external persons were present during the sessions besides the two researchers and the participants.*”  Lines 163-164: “*Both* sessions were recorded and lasted approximately one hour each*, and no participants dropped out of the sessions.*”  We added a date range of the literature search in the Methods section, lines 130-131 added; “*In October 2020,* we searched for previous research on ice cleats to supplement the model and analyze the plausibility of its assumptions, using combinations of the following keywords in PubMed and Scopus”  We added information about the interview guide used in the Supporting information section, line 617: “*S1 Appendix. Semi-structured interview guide. (DOCX)*”. |
| 3. We note that you have indicated that data from this study are available upon request. PLOS only allows data to be available upon request if there are legal or ethical restrictions on sharing data publicly. For information on unacceptable data access restrictions, please see http://journals.plos.org/plosone/s/data-availability#loc-unacceptable-data-access-restrictions.    In your revised cover letter, please address the following prompts:   a) If there are ethical or legal restrictions on sharing a de-identified data set, please explain them in detail (e.g., data contain potentially identifying or sensitive patient information) and who has imposed them (e.g., an ethics committee). Please also provide contact information for a data access committee, ethics committee, or other institutional body to which data requests may be sent. b) If there are no restrictions, please upload the minimal anonymized data set necessary to replicate your study findings as either Supporting Information files or to a stable, public repository and provide us with the relevant URLs, DOIs, or accession numbers. Please see http://www.bmj.com/content/340/bmj.c181.long for guidelines on how to de-identify and prepare clinical data for publication. For a list of acceptable repositories, please see http://journals.plos.org/plosone/s/data-availability#loc-recommended-repositories. | **Response to editor:**  Data Availability: The Swedish Ethical Review Authority has approved the study according to the Swedish Ethical Review Act (2003:460) regarding the ethical review of research involving humans. As dictated by the ethical body that approved the study and the promise to participants in their informed consent, the research data collected in the present study cannot be shared publicly as the data contain potentially identifying and sensitive personal data according to article 9 the General Data Protection Regulation (EU 2016/679), and public availability would compromise participant privacy. Data will be stored for at least 10 years at Karlstad University to enable review. Data are available for researchers who meet the criteria for access to confidential data. Data is covered by the Swedish Public Access to Information and Secrecy Act (2009:400) and a confidentiality assessment will be performed at each individual request. Permission from Karlstad University, Department of Political, Historical and Cultural Studies, has to be obtained before data can be accessed. Data requests may be sent to the Research data group at Karlstad University. Adress: Karlstad University, 651 88 Karlstad, Sweden. Tel. +46 54-700 10 00. E-mail: [forskningsdata@kau.se](mailto:forskningsdata@kau.se) |

Also, we would like to thank the reviewers for pointing out possible flaws in the manuscript. The reviewers’ comments point out that there are some clarifications needed to be added to the manuscript. We address them accordingly in the table below.

| **Reviewers’ comments to the Author** | **Authors’ response** |
| --- | --- |
| *Review Comments to the Author*  **Reviewer #1**: I recommend this manuscript , the ice cleat program showed a good results in protecting people specially the elderly in decreasing the risk of road injury during winter time.  And the Swedish municipal ice cleat distribution programs is targeting mostly the elderly people over 75 years to support them with ice cleat that will lead to more protection and lowering the risk of injury. | **Response to Reviewer#1**  Thank you for your taking the time to review our manuscript and for your positive feedback. |
| **Reviewer #2**: Clearly a great deal of effort went into this study, however a number of areas need clarifications:  - Were there set targets at the onset of the intervention. Measurements of the success will be best adjudged by how well these targets were achieved or otherwise e.g. did they target all potential users or did they seek to meet a certain percentage? The quoted figures in this report carry little weight in themselves if not in the context of these targets. | **Response to Reviewer#2**  The information that the municipalities gave us is unclear if they had set any predetermined set goals. We agree that this would give more context to the results and the manuscript overall. However, we try to address this issue by presenting interesting output from our data. The most indicative measure of success is defined by the number of ice cleats distributed divided by the number of targeted population living in the municipality, which overall was 40.5 percent. While the overall distribution, in terms of distributed ice cleats divided by the number of bought ice cleats, is about 90 percent for all programs. We agree that it would have been interesting to compare these numbers to a pre-determined set of goals within each municipality, but we also think that these quantitative measures of success (in terms of reach) also provide useful information in absence of a dichotomization of success.  **Revised text:**  We agree that it is imperative to inform the reader about the lack of data if the municipalities had predetermined goals with respect to reach. We clarify that such information was not given to us in the “Analysis of program reach” section, lines 111-112: “*To our knowledge, the municipalities did not set predetermined goals that can be used to define a successful ice cleat program.*” |
| **Reviewer #2**:  - How many people used or had access to the cleats before the intervention?  - What were the fall rates before and after the intervention? | **Response to Reviewer#2**  We agree that these are very important questions. However, it was beyond the scope of this study to examine how many people used or had access to ice cleats before the interventions were introduced, nor the fall rates before and after the intervention. In this paper, we set out to conduct a process evaluation and study the logic of these interventions from a more theoretical perspective in order to prepare for a full-scale impact evaluation study, as we discuss and suggest in the discussion section of the paper. In future studies, we will use what we learned in our process evaluation to conduct an impact evaluation of these interventions that investigate effects on fall injury rates.  **Revised text:**  We have attempted to clarify that our purpose was to conduct a process evaluation to study the implementation of ice cleat programs, and to highlight early that it is indeed our intention to try to answer these more quantitative outcome questions in upcoming studies. Specific changes:  Line number 69: changed the wording to avoid mentioning impact evaluations before the purpose to avoid confusion. The sentence now reads: “The utility of process evaluation and theoretical deconstruction of the logic behind a program have been emphasized in the program evaluation literature *as a means to study the implementation of public health interventions* [8-10].”  Lines 71-72, added; “The current paper aims to describe the implementation and characteristics of Swedish municipal ice cleat programs and analyze the logic of their design *by applying a summative process evaluation*”.  Lines 74-76, added: “*This study is a part of a project that aims to conduct a comprehensive evaluation of Swedish ice cleat distribution programs by assessing their implementation, impact, and cost-effectiveness*.”  Lines 80-82, added: “*The scope of the current paper is to investigate implementation and logic of ice cleat programs to provide a “qualitative flesh” to future impact evaluations, in which we will investigate their impacts on fall injury rates*.” |
